# Postmortem Sampling in Piglet Populations: Unveiling Specimens Accuracy for Porcine Reproductive and Respiratory Syndrome Detection

**DOI:** 10.3390/pathogens13080649

**Published:** 2024-08-02

**Authors:** Mariana Kikuti, Claudio Marcello Melini, Xiaomei Yue, Marie Culhane, Cesar A. Corzo

**Affiliations:** Department of Veterinary Population Medicine, University of Minnesota, Saint Paul, MN 55108, USA; mkikuti@umn.edu (M.K.); melin145@umn.edu (C.M.M.); yue00075@umn.edu (X.Y.); grame003@umn.edu (M.C.)

**Keywords:** postmortem sampling, PRRSV detection, specimen sensitivity, disease monitoring, diagnostic accuracy

## Abstract

Specimens collected from dead pigs are a welfare-friendly and cost-effective active surveillance. This study aimed to evaluate the accuracy of different postmortem specimens from dead piglets for disease detection, using PRRSV as an example. Three farrow-to-wean farms undergoing PRRSV elimination were conveniently selected. Samples were collected at approximately 8- and 20-weeks post-outbreak. Postmortem specimens included nasal (NS), oral (OS), and rectal (RS) swabs, tongue-tip fluids (TTF), superficial inguinal lymph nodes (SIL), and intracardiac blood. These were tested individually for PRRSV by RT-PCR. Sensitivity, specificity, negative and positive predictive values, and agreement of postmortem specimens were calculated using intracardiac sera as the gold standard. OS and SIL had the best overall performance, with sensitivities of 94.6–100%, specificities of 83.9–85.1%, and negative predictive values of 97.3–100%. TTF had high sensitivity (92.2%) but low specificity (53.9%) and positive predictive value (48.3%). While challenges in meeting sampling targets due to variable pre-weaning mortality were noted, PRRS was detected in all postmortem specimens. OS and NS showed promising results for disease monitoring, though TTF, despite their sensitivity, had lower specificity, making them less suitable for individual infection assessment but useful for assessing environmental contamination.

## 1. Introduction

Effective disease surveillance is crucial for safeguarding swine-herd health and preventing the epidemic transmission of diseases. By promptly detecting and monitoring infectious pathogens, surveillance plays an essential role in limiting the spread of diseases within swine populations, thereby preventing major economic repercussions. Surveillance not only enables the early identification of outbreaks and emerging pathogens but also facilitates informed control and intervention decisions. Consequently, it comprises the foundation of any comprehensive disease-control program, highlighting its role in ensuring the resilience and sustainability of swine production systems.

In the United States (U.S.), porcine reproductive and respiratory syndrome (PRRS) is one of the main health challenges faced by swine herds. Despite extensive efforts, including increasing herd-level immunity to reduce within-herd transmission and produce PRRS virus (PRRSV)-negative pigs [1,2], PRRS outbreaks continue to afflict a significant portion of U.S. breeding herds, causing a major economic impact [3,4]. The estimated annual PRRS incidence ranges around 20–30%, and the weekly PRRS virus prevalence was 20–40% between 2019 and 2023 in U.S. breeding herds [5,6].

Traditionally, PRRSV surveillance efforts in breeding herds have heavily relied upon antemortem sampling methods, including individual pig-level blood (serum), oropharyngeal, and nasal swabs, alongside group-level oral fluids and environmental samples [7]. Although the detection sensitivity of these methodologies varies, these antemortem specimens collected directly from individual pigs tend to have higher analytical sensitivity than the group or environmental specimens [8]. However, individual pig sampling often demands considerable labor and resources and diverts farm personnel from their routine tasks, posing logistical challenges, particularly in the face of the labor shortages prevalent in the industry. Certain individual sample-collection methods pose some level of occupational hazard as personnel can be injured in the process while restraining the pig or collecting the sample. Moreover, the practice can potentially cause momentary pain and distress to the animal, and attention to the restraint methods and volume collected is recommended [9]. Processing-fluid (e.g., serosanguineous fluid collected from the testicles/tails after castration and tail-docking practices) testing arose as a promising specimen for PRRS detection and monitoring of the neonatal population because they are easy and cost-effective to collect since these are routinely performed practices in the U.S. [7,10]. Routine tail-docking practices without evidence of tail-biting go against the European Union’s Council Directive [11], thus monitoring the dead piglet population through sampling tongue tips for PRRSV diagnosis was proposed as an alternative to processing-fluids surveillance for PRRS and was quickly adopted in the U.S. as a complementary specimen when monitoring breeding herds [12,13].

Even though postmortem specimens can be utilized for detecting a variety of swine diseases, they are typically used for diagnostic confirmation rather than for screening herds to ascertain herd disease status. Still, postmortem surveillance has been used most notably for African Swine Fever (ASF) and Classical Swine Fever (CSF) detection. Weekly sampling of dead post-weaning pigs was considered effective for early CSF detection in the absence of clinical suspicion of disease, lagging the infection in 14 to 30 days depending on the transmission rate of the virus [14]. For ASF, superficial inguinal lymph nodes have been proposed for screening dead pigs and were proven to highly correlate with detection in the spleen [15]. Thus, investigating practical, cost-effective, and animal-welfare-friendly postmortem specimens is essential to address challenges and discover new opportunities for disease diagnosis and surveillance in breeding herds.

This study aimed to compare pre-weaning mortality sampling to standard live piglet surveillance and determine the sensitivity of each postmortem specimen collected using PRRS as an example.

## 2. Materials and Methods

### 2.1. Study Design

Three U.S. farrow-to-wean farms undergoing a wild-type PRRS outbreak and that had decided to pursue elimination were conveniently selected. The eligibility criteria included farms reporting a PRRS outbreak from a provisionally negative or negative status to the Morrison Swine Health Monitoring Project (MSHMP). The MSHMP is a voluntary initiative where U.S. swine producers and their veterinarians share the weekly health status of their breeding herds, which account for approximately 60% of the U.S. breeding herd [16]. Under the eligibility criteria, farms would only be eligible to enroll if a PRRS outbreak was reported from a provisionally negative (negative breeding replacements are introduced and remain negative for at least 60 days) or negative (ELISA negative herd) status according to the American Association of Swine Veterinarian PRRS breeding-herd classification [17,18]. The exclusion criteria comprised farms with ongoing PRRS-modified live vaccination protocols for sows or piglets or farms where vaccination was planned as part of the PRRS outbreak intervention and elimination protocol. These criteria were in place to ensure that any viral detection during the study would be only associated with the wild-type PRRS outbreak.

Farm 1 and Farm 2 reported the PRRS outbreak on 14 December 2022, from a previously provisionally negative status, and on 4 January 2023, from a previous negative status, respectively. Farm 3 reported the PRRS outbreak on 11 October 2023, from a provisionally negative status. Farm 1 has an average inventory of 5000 sows, and the reported outbreak was associated with a Lineage 1C PRRSV2 (variant 1C.5). Farm 2 averages 2800 sows, and Farm 3 averages 2500 sows. Both outbreaks were associated with Lineage 1A PRRSV2 (variant 1A.2 and 1A.13, respectively). Lineages and variants [19,20,21,22,23] were assigned based on available ORF5 sequencing sent for routine diagnosis by the farms’ veterinarians. All farms are representative of modern pig production, have year-round negative-pressure air-filtration systems, and are located in the Midwestern U.S. Each farm was sampled at approximately 8 (Visit 1) and 20 weeks (Visit 2) after the outbreak detection, representing high-medium and medium-low within-herd PRRS prevalence. A total of 30 dead piglets sampled were targeted during Visit 1, which would provide 95% confidence to detect at least one PCR-positive pig when the within-herd prevalence is at least 10%. Likewise, a target of 60 dead piglets was set for Visit 2 to provide us with a 95% confidence of detecting PRRS at a 5% within-herd prevalence. Similarly, 30 and 60 live piglets were targeted at each sampling point. Live piglets were randomly chosen from rooms where the dead piglets originated. Although neither healthy nor sick piglets were specifically targeted, a mix of viremic and non-viremic piglets was expected, as sampling occurred when the prevalence was presumably high-medium and medium-low.

### 2.2. Sampling and Testing

Postmortem sampling of piglets found dead or euthanized according to farms’ regular animal care protocols was conducted. Postmortem specimens consisted of individual sterile swabs of the nasal, oral, and rectal cavities, tongue tips, superficial inguinal lymph nodes, and serum collected from intracardiac blood. The scissors and pliers used to collect tongue tips were cleaned with disinfectant wipes between each animal, and a new pair of gloves was used to handle each animal. Similarly, superficial inguinal lymph nodes were collected in Farm 3 using disposable scalpels while respecting the same efforts in avoiding cross-contamination as described for tongue tips. Swabs were collected using a BD BBL CultureSwab (Cat. No. L4320116, BD, Franklin Lakes, NJ, USA) containing liquid Stewart medium, liquid Amies medium, and Cary–Blair agar gel medium. Sera were collected in BD Vacutainer 8.5 mL tubes (Cat. No. 367988, BD, Franklin Lakes, NJ, USA). Tongue tips and lymph nodes were individually placed in Whirl-Pack bags. No RNA stabilizer solution was added to any sample. All specimens were submitted to the University of Minnesota Veterinary Diagnostic Laboratory (VDL) for individual PRRSV RT-PCR testing. Tongue tips were first processed individually by adapting a previously described methodology [12]. Briefly, tongue tips were frozen at −20 °C for at least 8 h and thawed. Subsequently, 500 µL of phosphate-buffered saline solution (PBS, pH 7.4, Cat. No. 10010023, Thermo Fischer Scientific, Waltham, MA, USA) were added to each bag containing the tissue, followed by manual homogenization for one minute. Tongue-tip fluids were submitted for individual PRRSV RT-PCR testing. Likewise, superficial inguinal lymph nodes (SIL) were submitted as tissue for individual PRRSV RT-PCR testing. At the VDL, a 20% tissue −80% Hanks solution homogenate was prepared with SIL, and all samples underwent a high throughput total nucleic acid extraction. PRRSV RT-PCR is performed using ThermoFisher VetMAX™ PRRSV EU & NA 2.0 Kit (Cat No. A35751, Thermo Fischer Scientific, Waltham, MA, USA).

Live piglet blood sampling was conducted preferentially from rooms from which the dead pigs originated via jugular venipuncture. Sera from live piglets were tested for PRRSV by RT-PCR in pools of five; any sera from positive pools were then tested individually. Additionally, all positive oral swabs, tongue-tip fluids, and serum collected from dead piglets at Farm 3 Visit 1 were individually submitted for PRRSV ORF5 sequencing.

### 2.3. Data Analysis

Sera from live piglets were used to estimate the within-herd PRRS prevalence using the current industry monitoring sampling approach. The sensitivity, specificity, positive and negative predictive values, and the agreement of tongue-tip fluids, superficial inguinal lymph nodes, and oral, nasal, and rectal swabs were calculated using the postmortem intracardiac sera as the assumed gold standard. Exact binomial confidence intervals for the sensitivity, specificity, and positive and negative predictive values were estimated. Agreement between each specimen and intracardiac sera, as well as agreement between tongue-tip fluids and oral swabs, were calculated. The analysis was performed using STATA 18 [24]. The success rate in obtaining a PRRSV sequence in oral swabs, tongue-tip fluids, and intracardiac sera was calculated as the percent of RT-PCR positive samples in which an ORF5 PRRSV sequence was successfully recovered, and the percent nucleotide identity between samples was described.

### 2.4. Ethics Statement

This study was approved by the University of Minnesota Institutional Animal Care and Use Committee under protocol ID: 2208-40341A.

## 3. Results

The live piglet sampling target was achieved at all sampling points, except for Farm 1 Visit 1 (F1V1), where one sample yielded an insufficient serum volume for testing. In contrast, the postmortem sampling target was only met in F1V1 and Farm 3 Visit 2 (F3V2), although one sample from F3V2 also had an insufficient serum volume for testing. Overall, PRRSV was detected by RT-PCR in all specimens and all farm visits except for Farm 2 Visit 2 (F2V2) and F3V2, in which PRRSV was not detected in the swabs and intracardiac sera, respectively (Table 1). The estimated prevalence was 79.31% (95% CI 60.3–92.0%) in F1V1, 63.33% (95% CI 43.9–80.1%) in Farm 2 Visit 1 (F2V1), 30.00% (95% CI 14.73–49.40%) in Farm 3 Visit 1 (F3V1), 10.00% (95% CI 3.8–20.5%) in Farm 1 Visit 2 (F1V2), 0.00% (95% CI 0.0–6.0%) in F2V2, and 10.00% (95% CI 4.66–20.15%) in F3V2. Meanwhile, in the postmortem samples, PRRSV was detected in 0% (in oral, nasal, and rectal swabs from F2V2) to 100% (in tongue-tip fluids from F3V1).

Overall, the agreements between each postmortem specimen type and postmortem sera were 87.10% for oral swabs (kappa 0.72, *p* < 0.001), 85.48% for nasal swabs (kappa 0.68, *p* < 0.001), 84.95% for rectal swabs (kappa 0.66, *p* < 0.001), 67.39% for tongue-tip fluids (kappa 0.40, *p* < 0.001), and 88.89% for superficial inguinal lymph nodes (kappa 0.74, *p* < 0.001) (Table S1). The agreement between tongue-tip fluids and oral swab samples was 78.24% (kappa 0.58, *p* < 0.001). The overall sensitivity ranged from 85.71% for rectal swabs to 100.00% for superficial inguinal lymph nodes (Table 2). However, the overall specificity ranged from 53.91% in tongue-tip fluids to 85.07% for superficial inguinal lymph nodes. Both sensitivity and specificity varied between sampling points, with the lowest sensitivity found in F2V2 for oral, nasal, and rectal swabs, while the lowest specificity was found in F3V1 for tongue-tip fluids. Positive and negative predictive values are also shown in Table 2, with tongue-tip fluids having an overall lower positive predictive value (PPV) and higher negative predictive value (NPV). The accuracy by sampling day and age categories is displayed in Tables S2 and S3.

Positive intracardiac serum samples had a median Ct of 20.76, while the median Ct values for oral, nasal, and rectal swabs were 29.13, 30.07, and 30.98, respectively. The median Ct value for positive tongue-tip fluids was 27.64, while the median Ct for positive superficial inguinal lymph nodes was 21.42. Of the 31 tongue-tip fluids that were RT-PCR positive in F3V1, a PRRSV ORF5 sequence was obtained for 29 of them (median Ct 23.04), yielding a 93.5% sequencing success rate in this specimen. Similarly, the sequencing success rates among the RT-PCR positive oral swabs and intracardiac serum were 82.1% (23/28, median Ct 25.02) and 91.3% (21/23, median Ct 20.38), respectively (Table S4). The minimum percent nucleotide identity between all the PRRSV ORF5 sequences generated in F3V1 was 99.2%, suggesting very low within-farm diversity. However, for 12 of the 19 animals from which sequences were successfully obtained from all three specimens (serum, tongue-tip fluid, and oral swab), no nucleotide differences between specimens were found. On the other hand, four animals yielded identical sequences in the tongue-tip fluid and serum, with up to two nucleotide differences found in oral swabs. One animal yielded identical sequences in the tongue-tip fluid and oral swab, with one nucleotide difference in the sequence yielded from serum. Lastly, for two animals, up to two nucleotide differences were found between all three specimens.

## 4. Discussion

While the serum from the live piglets sampling target was easily met, the same cannot be said about the postmortem sampling target. In this study, one-day farm visits were proposed, allowing for the postmortem sampling of piglets that were found dead or euthanized within the previous 24–48 h and still present at the farm. However, although the reported pre-weaning mortality in herds undergoing a PRRS outbreak can be as high as 100% in particularly severe cases [25], several studies report pre-weaning mortality around 20–50% when herds are facing a PRRS outbreak [26,27,28]. This highly variable parameter likely depends on secondary factors, such as disease management, PRRSV variant, herd immunity, co-infections, and timing within the outbreak (whether at the beginning, when sows have been recently exposed to the newly introduced virus, or towards the end when immunity has likely developed and the herd is close to stability). This was illustrated in a recent study in which piglet mortality was 33%, 22%, and 19% at 1.5, 8, and 12 months after the PRRSV-1 outbreak onset [29]. Thus, although postmortem sampling can be a welfare-friendly alternative for disease monitoring, it can be challenging to obtain appropriate sample sizes in short periods if statistical power is needed to respond to specific scientific questions. Still, the detection of PRRS at all sampling points in at least one of the specimens assessed was possible.

The within-herd prevalence needs to be interpreted with caution, considering the sampling time, specimen, and sample size. Serum from live piglets was used as a proxy of within-herd prevalence. Although initially intending to sample at high/medium and medium/low prevalence, sampling at 55–63 days post-onset of the outbreak yielded relatively high estimated prevalences on farms 1 and 2 of 79.31% and 63.33%. However, sampling at 140 days post-onset of the outbreak yielded a very low estimated prevalence at 0–10%. An important consideration when interpreting the estimated prevalence is that sample size was calculated to estimate freedom of disease (or the probability of detecting at least one positive animal) instead of within-herd prevalence. This strategy is commonly used in the field to ascertain if a herd has reached stability, aiming to sample due to wean piglets, and is often extrapolated to an estimate of within-herd prevalence, since a proper sample size could be too costly for daily operations. Although prevalence does not directly affect a test’s diagnostic accuracy, an unbalanced population (e.g., mostly healthy or mostly diseased) can result in wide confidence intervals, hindering interpretation. However, this was designed as a pilot study, so the information generated here could help inform sample-size calculations for future studies. Moreover, the positivity rate in live piglets did not directly correspond to the positivity rate in the intracardiac sera of dead piglets. At some sampling points, the positivity rate was higher in live piglets (e.g., F1V1) while in others, it was higher in dead piglets (e.g., F3V1). Thus, the positivity rate in postmortem sampling might not accurately represent within-herd prevalence.

PRRS was detected in all the postmortem specimens assessed at least once, demonstrating the viability of these specimens for PRRS monitoring with different levels of confidence. Overall, the highest agreement of postmortem intracardiac sera was with superficial inguinal lymph nodes, followed by oral and nasal swabs, also presenting the highest overall sensitivity and specificity. However, the superficial lymph node was only assessed in Farm 3 due to the project receiving additional funding, allowing for the inclusion of this specimen. The wider accuracy confidence intervals should be reflective of this specimen’s overall performance since PRRSV detection is not dependent on farm-specific characteristics. However, a more in-depth assessment with larger sample sizes is still necessary to more precisely assess this specimen’s accuracy. Although tongue-tip fluids had good sensitivity, their specificity was overall low. The PRRSV1 positivity rate has also been reported to be higher than fetal serum and thymus [30]. Although the authors suggest this represents that tongue-tip fluids are suited for detecting vertical transmission, PRRS viremia is expected to last weeks to months in animals infected in-uterus or at younger ages [27,31]. Thus, it is likely that those represent false-positive results. False positives in tongue-tip fluids could be attributed to a myriad of explanations. For this study, given the low probability of contamination during sample handling under laboratory settings and the fact that positive and false-positive samples were not handled sequentially, along with the practice of researchers changing gloves and disinfecting materials between each sample collection, it is reasonable to assume that the false positives may represent within-farm environmental contamination. This could either be related to live piglets interacting with the dead animals before they are removed from the farrowing pens or during the farm’s regular handling and storing of dead animals until sampling. The amount of false-positive tongue-tip fluids was reflected as an overall lower positive predictive value of tongue-tip fluids, with only a 48% probability of actually being viremic given a positive RT-PCR result, indicating they might not be ideal specimens for the individual assessment of infection amongst dead piglets. Interestingly, oral swabs had a higher accuracy than tongue tips, and agreement between both specimens was just under 80%. One possible reason could be that tongue-tip collection involves greater manipulation of the animal and increased exposure to environmental contaminants, especially if the animal’s tongue protrudes after death. In contrast, oral swab collection entailed sampling areas between the teeth and inner cheek, as well as beneath the tongue, which are less susceptible to environmental contaminants. Rectal swab performance was only slightly worse than oral and nasal swabs. However, this result was somewhat expected since the presence of inhibitors in fecal samples might hinder RNA detection [32]. Moreover, viral isolation from the feces of PRRSV-infected animals has been reported as infrequent [33,34], suggesting potential infrequent shedding through this route, as well as highlighting the difficulties in PRRSV diagnosis with this specimen. Although false positives might be an issue when trying to assess disease prevalence amongst the mortality, having highly sensitive detection methods, even those that detect environmental contamination, might represent an improvement in detecting the presence of the virus on a farm. It is important to note, however, that false positives in the context of the accuracy calculation described here represent positive RT-PCR results when animals were not viremic. However, these likely represent correctly ascertained positive samples, in the sense that genetic material was present.

The specimens collected for PRRSV surveillance might also need to serve the purpose of PRRSV sequencing to reduce costs while generating valuable information for molecular epidemiology investigations. Here, three specimens were selected to investigate the success rate in obtaining a PRRSV ORF5 sequence. These were selected based on their routine use in the field (tongue-tip fluids), ease of collection (oral swabs), and lowest probability of environmental contamination (intracardiac serum). Even though the sequencing success rate was higher in tongue-tip fluids, all three specimens presented a good sequencing success rate (82.1% to 93.5%), indicating that sequencing was not a limitation for any of them. The overall viral diversity was found to be low based on the F3V1 PRRSV ORF5 sequencing from dead piglets. Still, discrepancies between sequences generated from different specimens from the same animal were found. These usually comprised one to two nucleotide differences but resulted in one to two amino acid changes in the samples collected from three animals. This can be relevant for within-farm PRRSV diversity studies.

Additional factors that need to be considered when choosing a postmortem sampling method include ease of collection, workers’ safety, and cost. While intracardiac serum and superficial inguinal lymph nodes would be ideal specimens to address most research and epidemiological questions, they require personnel training for proper and safe sample collection. It also requires investments in supplies, such as individual needles, syringes, and tubes for intracardiac blood collection or tweezers and scalpels for superficial inguinal lymph nodes. Collecting lymph nodes with a knife is possible, but can be challenging in smaller neonatal dead animals, potentially posing a worker safety concern. Still, both methods could be appropriate even for foreign disease monitoring, such as African Swine Fever, since they can be collected without any or with minimal blood spilling. Tongue tips are easy specimens to collect but also require some manipulation of the animals with sharps, which can result in longer personnel time dedicated to sample collection when compared to swabs. In terms of cost, tongue tips might be the least costly specimen, since they can be collected with just a knife and are typically tested in pools or as a unique aggregated sample representing the farm [12,13]. Still, this specimen requires minimal training and can easily be adopted on any farm. Lastly, swabs (whether oral, nasal, or rectal) are quick, safe, and easy to collect, requiring minimal training. However, the costs associated with this specimen’s collection might be higher, since it requires investments in individual swabs. Litter-level pooling of oral and nasal swabs seems to have a high probability of detecting PRRSV when pools are comprised of at least a 26% proportion of positives [35], but further studies on its diagnostic performance for PRRS detection are still needed.

## 5. Conclusions

Overall, this study provides insight into postmortem sampling as a welfare-friendly alternative for disease monitoring in breeding herds, using PRRS as an example. Because postmortem intracardiac blood collection requires training, oral and nasal swabs are promising specimens with good sensitivity and specificity. While tongue-tip fluids presented good sensitivity, their specificity and positive predictive values were low, demonstrating they are likely not ideal for individual infection assessment, although they might still be useful in assessing environmental contamination within a farm.

## Figures and Tables

**Table 1:** PRRSV RT-PCR positivity by farm, sampling point according to days post-outbreak, and specimen types.

	Farm 1 Visit 1 (F1V1)	Farm 1 Visit 2 (F1V2)	Farm 2 Visit 1 (F2V1)	Farm 2 Visit 2 (F2V2)	Farm 3 Visit 1 (F3V1)	Farm 3 Visit 2 (F3V2)
Days post-outbreak	63	140	55	140	56	147
Live piglets						
Serum	23/29 (79.31%)	6/60(10.00%)	19/30 (63.33%)	0/60 (0.00%)	9/30 (30.00%)	6/60 (10.00%)
Post-mortem sampling						
Serum	14/30 (46.67%)	6/33 (18.18%)	12/16 (75.00%)	1/17 (5.88%)	23/31 (74.19%)	0/59 (0.00%)
OS	15/30 (50.00%)	9/38 (23.68%)	15/17 (88.24%)	0/20 (0.00%)	28/31 (90.32%)	9/60 (15.00%)
NS	14/30 (46.67%)	7/38 (18.42%)	14/17 (82.35%)	0/20 (0.00%)	28/31 (90.32%)	10/60 (16.67%)
RS	13/30 (43.33%)	5/38 (13.16%)	12/17 (70.59%)	0/20 (0.00%)	28/31 (90.32%)	12/60 (20.00%)
TTF	25/30 (83.33%)	21/36 (58.33%)	15/17 (88.24%)	2/19 (10.53%)	31/31 (100.00%)	24/60 (40.00%)
SIL	NA	NA	NA	NA	26/31 (83.87%)	7/60 (11.67%)

OS: Oral swab; NS: nasal swab; RS: rectal swab; TTF: tongue-tip fluid; SIL: superficial inguinal lymph node; NA: not available.

**Table 2:** Accuracy of different postmortem specimen types considering their correspondent postmortem serum as a gold standard.

	TP	FN	FP	TN	Sensitivity (95% CI)	Specificity (95% CI)	PPV (95% CI)	NPV (95% CI)
OS	53	3	21	109	94.64% (85.13–98.88%)	83.85% (76.37–89.71%)	71.62% (59.95–81.50%)	97.32% (92.37–99.44%)
NS	50	6	21	109	89.29% (78.12–95.97%)	83.85% (76.37–89.71%)	70.42% (58.41–80.67%)	94.78% (88.99–98.06%)
RS	48	8	20	110	85.71% (73.78–93.62%)	84.62% (77.24–90.34%)	70.59% (58.29–81.02%)	93.22% (87.08–97.03%)
TTF	55	1	59	69	98.21% (90.45–99.95%)	53.91% (44.88–62.75%)	48.25% (38.79–57.80%)	98.57% (92.30–99.96%)
SIL	23	0	10	57	100.00% (85.18–100.00%)	85.07% (74.26–92.60%)	69.70% (51.29–84.41%)	100.00% (93.73–100.00%)

TP: true positive, FN: false negative, FP: false positive, TN: true negative, CI: confidence interval, OS: oral swab, NS: nasal swab, RS: rectal swab, TTF: tongue-tip fluid, SIL: superficial inguinal lymph node, PPV: positive predictive value, NPV: negative predictive value, NA: not available.

## Data Availability

Summarized data to calculate sensitivity, specificity, and positive and negative predictive values are included in this published article and its supplementary information files. Additional data that support the findings of this study are available from the corresponding author upon reasonable request.

## Supplementary Materials

The following supporting information can be downloaded at https://www.mdpi.com/article/10.3390/pathogens13080649/s1: Table S1: Agreement between specimens by sampling point; Table S2: Accuracy of different postmortem specimen types considering their correspondent postmortem serum as a gold standard by sampling day. Table S3: Accuracy of different postmortem specimen types by age category considering their correspondent postmortem serum as a gold standard. Table S4: PRRSV RT-PCR Ct value and ORF5 sequencing success by specimen in animals that yielded at least one RT-PCR result.
