# Supplementary material for: Postmortem Sampling in Piglet Populations: Unveiling Specimens Accuracy for Porcine Reproductive and Respiratory Syndrome Detection
Source: Pathogens. 2024 Aug 2;13(8):649. doi: 10.3390/pathogens13080649 (PMC11356954; doi:10.3390/pathogens13080649)
Supplement: Supplementary file 1 [file pathogens-13-00649-s001.zip › 2024 07 22 Table S2.pdf]

Table S2. Accuracy of different postmortem specimen types considering their correspondent postmortem serum as a gold standard by sampling day.

	TP	FN	FP	TN	Sensitivity (95% CI)	Specificity (95% CI)	PPV (95% CI)	NPV (95% CI)
Farm 1 Visit 1								
OS	14	0	1	15	100.0% (76.8% - 100.0%)	93.8% (69.8% - 99.8%)	93.3% (68.1% - 99.8%)	100.0% (78.2% - 100.0%)
NS	13	1	1	15	92.9% (66.1% - 99.8%)	93.8% (69.8% - 99.8%)	92.9% (66.1% - 99.8%)	93.8% (69.8% - 99.8%)
RS	12	2	1	15	85.7% (57.2% - 98.2%)	93.8% (69.8% - 99.8%)	92.3% (64.0% - 99.8%)	88.2% (63.6% - 98.5%)
TTF	14	0	11	5	100.0% (76.8% - 100.0%)	31.3% (11.0% - 58.7%)	56.0% (34.9% - 75.6%)	100.0% (47.8% - 100.0%)
Farm 2 Visit 1								
OS	11	1	3	1	91.7% (61.5% - 99.8%)	25.0% (0.63% - 80.6%)	78.6% (49.2% - 95.3%)	50.0% (1.26% - 98.7%)
NS	11	1	2	2	91.7% (61.5% - 99.8%)	50.0% (6.76% - 93.2%)	84.6% (54.6% - 98.1%)	66.7% (9.43% - 99.2%)
RS	11	1	0	4	91.7% (61.5% - 99.8%)	100.0% (39.8% - 100.0%)	100.0% (71.5% - 100.0%)	80.0% (28.4% - 99.5%)
TTF	11	1	3	1	91.7% (61.5% - 99.8%)	25.0% (0.63% - 80.6%)	78.6% (49.2% - 95.3%)	50.0% (1.26% - 98.7%)
Farm 3 Visit 1								
OS	23	0	5	3	100.00% (85.18% - 100.00%)	37.50% (8.52% - 75.51%)	82.14% (63.11% - 93.94%)	100.00% (29.24% - 100.00%)
NS	22	1	6	2	95.65% (78.05% - 99.89%)	25.00% (3.19% - 65.09%)	78.57% (59.05% - 91.70%)	66.67% (9.43% - 99.16%)
RS	23	0	5	3	100.00% (85.18% - 100.00%)	37.50% (8.52% - 75.51%)	82.14% (63.11% - 93.94%)	100.00% (29.24% - 100.00%)
TTF	23	0	8	0	100.00% (85.18% - 100.00%)	0.00% (0.00% - 36.94%)	74.19% (55.39% - 88.14%)	NA
SIL	23	0	3	5	100.00% (85.18% - 100.00%)	62.50% (24.49% - 91.48%)	88.46% (69.85% - 97.55%)	100.00% (47.82% - 100.00%)
Farm 1 Visit 2								
OS	5	1	3	24	83.3% (35.9% - 99.6%)	88.9% (70.8% - 97.6%)	65.5% (24.5% - 91.5%)	96.0% (79.6% - 99.9%)
NS	4	2	2	25	66.7% (22.3% - 95.7%)	92.6% (75.7% - 99.1%)	66.7% (22.3% - 95.7%)	92.6% (75.7% - 99.1%)
RS	2	4	2	25	33.3% (4.33% - 77.7%)	92.6% (75.7% - 99.1%)	50.0% (6.76% - 93.2%)	86.2% (68.3% - 96.1%)
TTF	6	0	12	14	100.0% (54.1% - 100.0%)	53.8% (33.4% - 73.4%)	33.3% (13.3% - 59.0%)	100.0% (76.8% - 100.0%)
Farm 2 Visit 2								
OS	0	1	0	16	0.00% (0.00% - 97.5%)	100.0% (79.4% - 100.0%)	NA	94.1% (71.3% - 99.9%)
NS	0	1	0	16	0.00% (0.00% - 97.5%)	100.0% (79.4% - 100.0%)	NA	94.1% (71.3% - 99.9%)
RS	0	1	0	16	0.00% (0.00% - 97.5%)	100.0% (79.4% - 100.0%)	NA	94.1% (71.3% - 99.9%)
TTF	1	0	1	14	100.0% (2.5% - 100.0%)	93.3% (68.1% - 99.8%)	50.0% (1.26% - 98.7%)	100.0% (76.8% - 100.0%)
Farm 3 Visit 2								

OS	0	0	9	50	NA	84.75% (73.01% - 92.78%)	0.00% (0.00% - 33.63%)	100.00% (92.89% - 100.00%)
NS	0	0	10	49	NA	83.05% (71.03% - 91.56%)	0.00% (0.00% - 30.85%)	100.00% (92.75% - 100.00%)
RS	0	0	12	47	NA	79.66% (67.17% - 89.02%)	0.00% (0.00% - 26.46%)	100.00% (92.45% - 100.00%)
TTF	0	0	24	35	NA	59.32% (45.75% - 71.93%)	0.00% (0.00% - 14.25%)	100.00% (90.00% - 100.00%)
SIL	0	0	7	52	NA	88.14% (77.07% - 95.09%)	0.00% (0.00% - 40.96%)	100.00% (93.15% - 100.00%)

TP: True positive, FN: False Negative, FP: False positive, TN: True Negative, CI: Confidence interval, OS: Oral swab, NS: Nasal swab, RS: Rectal swab, TTF: Tongue tip fluid, SIL: Superficial inguinal lymph node, PPV: Positive predictive value, NPV: Negative predictive value, NA: not available.
