# Supplementary material for: Postmortem Sampling in Piglet Populations: Unveiling Specimens Accuracy for Porcine Reproductive and Respiratory Syndrome Detection
Source: Pathogens. 2024 Aug 2;13(8):649. doi: 10.3390/pathogens13080649 (PMC11356954; doi:10.3390/pathogens13080649)
Supplement: Supplementary file 1 [file pathogens-13-00649-s001.zip › 2024 07 22 Table S3.pdf]

Table S3. Accuracy of different postmortem specimen types by age category considering their correspondent postmortem serum as a gold standard.

	TP	FN	FP	TN	Sensitivity (95% CI)	Specificity (95% CI)	PPV (95% CI)	NPV (95% CI)
Stillborn (n=52)								
Oral swab	1	1	9	41	50.00% (1.26% - 98.70%)	82.00% (68.60% - 91.40%)	10.00% (0.25% - 44.50%)	97.60% (87.40% - 99.90%)
Nasal swab	1	1	11	39	50.00% (1.26% - 98.70%)	78.00% (64.00% - 88.50%)	8.33% (0.21% - 38.5%)	97.50% (86.80% - 99.90%)
Rectal swab	1	1	13	37	50.00% (1.26% - 98.70%)	74.00% (59.70% - 85.40%)	7.14% (0.18% - 33.90%)	97.40% (86.20% - 99.90%)
Tongue tip	2	0	21	29	100.00% (15.80% - 100.00%)	58.00% (43.20% - 71.80%)	8.70% (1.07% - 28.00%)	100.00% (88.10% - 100.00%)
SIL	1	0	7	37	100.00% (2.50% - 100.00%)	84.10% (69.90% - 93.40%)	12.50% (0.32% - 52.70%)	100.00% (90.50% - 100.00%)
1 to 7 days old (n=86)								
Oral swab	23	1	7	55	95.80% (78.90% - 99.90%)	88.70% (78.10% - 95.30%)	76.70% (57.70% - 90.10%)	98.20% (90.40% - 100.00%)
Nasal swab	22	2	8	54	91.70% (73.00% - 99.00%)	87.10% (76.1% - 94.30%)	73.30% (54.10% - 87.70%)	96.40% (87.70% - 99.60%)
Rectal swab	22	2	3	59	91.70% (73.00% - 99.00%)	95.20% (86.50% - 99.00%)	88.00% (68.80% -97.50%)	96.70% (88.70% - 99.60%)
Tongue tip	23	1	26	34	95.80% (78.90% - 99.90%)	56.70% (43.20% - 69.40%)	46.90% (32.50% - 61.70%)	97.10% (85.10% - 99.90%)
SIL	9	0	1	17	100.00% (66.40% - 100.00%)	94.40% (72.70% - 99.90%)	90.00% (55.50% - 99.70%)	100.00% (80.50% - 100.00%)
8 to 22 days old (n=44)								
Oral swab	28	1	3	12	96.60% (82.20% - 99.90%)	80.00% (51.90% - 95.70%)	90.30% (74.20% - 98.00%)	92.30% (64.00% - 99.80%)
Nasal swab	26	3	0	15	89.70% (72.60% - 97.80%)	100.00% (78.20% - 100.00%)	100.00% (86.80% - 100.00%)	83.30% (58.60% - 96.40%)
Rectal swab	24	5	2	13	82.80% (64.20% - 94.20%)	86.70% (59.50% - 98.30%)	92.30% (74.90% - 99.10%)	72.20% (46.50% - 90.30%)
Tongue tip	29	0	10	5	100.00% (88.10% - 100.00%)	33.30% (11.80% - 61.6%)	74.40% (57.90% - 87.00%)	100.00% (47.80% - 100.00%)
SIL	13	0	0	2	100.00% (75.30% - 100.00%)	100.00% (15.80% - 100.00%)	100.00% (75.30% - 100.00%)	100.00% (15.80% - 100.00%)

TP: True positive, FN: False Negative, FP: False positive, TN: True Negative, CI: Confidence interval, SIL: Superficial inguinal lymph node, PPV: Positive predictive value, NPV: Negative predictive value, NA: not available.
