# Supplementary material for: Postmortem Sampling in Piglet Populations: Unveiling Specimens Accuracy for Porcine Reproductive and Respiratory Syndrome Detection
Source: Pathogens. 2024 Aug 2;13(8):649. doi: 10.3390/pathogens13080649 (PMC11356954; doi:10.3390/pathogens13080649)
Supplement: Supplementary file 1 [file pathogens-13-00649-s001.zip › 2024 07 24 Table S4.pdf]

Table S4. PRRSV RT-PCR Ct value and ORF5 sequencing success by specimen in animals that yielded at least one RT-PCR result.

Farm	Visit	Animal ID	Serum Ct value	Oral Swab Ct value	Tongue tip fluid Ct value	ORF5 sequence from Serum	ORF5 sequence from Oral swab	ORF5 sequence from Tongue tip fluid
1	1	2	Negative	Negative	28.81	Not tested	Not tested	Not tested
1	1	3	24.29	32.24	26.37	Not tested	Not tested	Not tested
1	1	4	10.81	25.64	18.92	Not tested	Not tested	Not tested
1	1	5	Negative	Negative	31.23	Not tested	Not tested	Not tested
1	1	7	Negative	Negative	33.22	Not tested	Not tested	Not tested
1	1	8	Negative	Negative	34.03	Not tested	Not tested	Not tested
1	1	9	26	30.62	23.94	Not tested	Not tested	Not tested
1	1	10	Negative	30.07	27.9	Not tested	Not tested	Not tested
1	1	11	12.63	24.66	19.47	Not tested	Not tested	Not tested
1	1	12	Negative	Negative	30.99	Not tested	Not tested	Not tested
1	1	15	23.12	24.05	25.26	Not tested	Not tested	Not tested
1	1	16	15.35	29.79	19.92	Not tested	Not tested	Not tested
1	1	17	16.05	28.39	21.13	Not tested	Not tested	Not tested
1	1	18	Negative	Negative	33.19	Not tested	Not tested	Not tested
1	1	20	Negative	Negative	35.05	Not tested	Not tested	Not tested
1	1	21	12.03	26.27	17.64	Not tested	Not tested	Not tested
1	1	22	Negative	Negative	32.83	Not tested	Not tested	Not tested
1	1	23	19.46	28.02	23.63	Not tested	Not tested	Not tested
1	1	24	18.29	28.13	22.53	Not tested	Not tested	Not tested
1	1	25	Negative	Negative	33.73	Not tested	Not tested	Not tested
1	1	26	17.41	26.08	21.73	Not tested	Not tested	Not tested
1	1	27	27.64	32.99	31.49	Not tested	Not tested	Not tested
1	1	28	Negative	Negative	33.3	Not tested	Not tested	Not tested
1	1	29	24.02	27.85	24.87	Not tested	Not tested	Not tested
1	1	30	21.31	29.28	24.31	Not tested	Not tested	Not tested
1	2	61	Negative	33.16	32.19	Not tested	Not tested	Not tested
1	2	65	Negative	31.24	32.22	Not tested	Not tested	Not tested
1	2	66	Negative	34.33	32.01	Not tested	Not tested	Not tested
1	2	67	33.34	31.22	30.23	Not tested	Not tested	Not tested
1	2	68	Negative	Negative	31.35	Not tested	Not tested	Not tested
1	2	69	Negative	Negative	32.56	Not tested	Not tested	Not tested
1	2	70	20.62	29.97	21.01	Not tested	Not tested	Not tested
1	2	71	32.63	24.05	19.42	Not tested	Not tested	Not tested
1	2	72	25.74	27.72	24.34	Not tested	Not tested	Not tested
1	2	73	30.33	Negative	34.04	Not tested	Not tested	Not tested
1	2	78	Negative	25.35	30.57	Not tested	Not tested	Not tested
1	2	79	Negative	Negative	35.07	Not tested	Not tested	Not tested

1	2	80	Negative	Negative	31.31	Not tested	Not tested	Not tested
1	2	81	Negative	Negative	37.09	Not tested	Not tested	Not tested
1	2	83	Negative	Negative	32.27	Not tested	Not tested	Not tested
1	2	84	Negative	Negative	31.15	Not tested	Not tested	Not tested
1	2	88	Negative	Negative	35.33	Not tested	Not tested	Not tested
1	2	89	Negative	Negative	35.32	Not tested	Not tested	Not tested
1	2	90	22.04	29.42	22.3	Not tested	Not tested	Not tested
1	2	91	Negative	Negative	33.35	Not tested	Not tested	Not tested
1	2	95	Negative	Negative	35.98	Not tested	Not tested	Not tested
2	1	61	20.9026	27.2463	21.0606	Not tested	Not tested	Not tested
2	1	62	12.1377	17.5121	18.8459	Not tested	Not tested	Not tested
2	1	63	19.0624	28.6101	24.0657	Not tested	Not tested	Not tested
2	1	64	Negative	24.6079	17.5669	Not tested	Not tested	Not tested
2	1	65	16.8901	29.2887	22.9556	Not tested	Not tested	Not tested
2	1	66	Negative	34.7876	25.855	Not tested	Not tested	Not tested
2	1	67	Negative	36.0469	28.0925	Not tested	Not tested	Not tested
2	1	68	22.2202	30.6545	27.0836	Not tested	Not tested	Not tested
2	1	69	15.9785	29.3575	20.2597	Not tested	Not tested	Not tested
2	1	70	20.4596	Negative	Negative	Not tested	Not tested	Not tested
2	1	71	34.9229	29.1924	24.8803	Not tested	Not tested	Not tested
2	1	72	16.1191	33.0046	25.1186	Not tested	Not tested	Not tested
2	1	73	18.0521	28.0352	20.8478	Not tested	Not tested	Not tested
2	1	74	Negative	34	32.3465	Not tested	Not tested	Not tested
2	1	76	23.1951	30.0435	25.097	Not tested	Not tested	Not tested
2	1	77	18.133	28.6733	22.1596	Not tested	Not tested	Not tested
2	2	222	Negative	Negative	36.88	Not tested	Not tested	Not tested
2	2	235	30.69	Negative	35.92	Not tested	Not tested	Not tested
3	1	271	Negative	Negative	28.91	Not tested	Not tested	Successful
3	1	272	20.1215	29.694	25.2	Successful	Successful	Successful
3	1	273	Negative	35.3305	32.04	Not tested	Not successful	Successful
3	1	274	Negative	36.33	30.72	Not tested	Not successful	Successful
3	1	275	12.9046	21.0603	18.93	Successful	Successful	Successful
3	1	276	16.6496	23.0833	19.78	Successful	Successful	Successful
3	1	277	Negative	Negative	32.11	Not tested	Not tested	Not successful
3	1	278	21.1851	21.0002	23.2	Successful	Successful	Successful
3	1	279	Negative	23.4891	21.07	Not tested	Successful	Successful
3	1	280	21.532	27.1249	21.19	Successful	Successful	Successful
3	1	281	16.2034	21.0067	20.1	Successful	Successful	Successful
3	1	282	Negative	25.0184	26.25	Not tested	Successful	Successful
3	1	283	21.2038	29.0074	22.49	Successful	Successful	Successful
3	1	284	17.6998	27.836	23.6	Successful	Successful	Successful

3	1	285	17.8545	24.6883	21.03	Successful	Successful	Successful
3	1	286	25.009	25.0227	23.76	Successful	Successful	Successful
3	1	287	19.34	26.1992	21.78	Successful	Successful	Successful
3	1	288	20.9	24.1042	21.6	Not successful	Successful	Successful
3	1	289	21.4263	31.1443	21.02	Successful	Successful	Successful
3	1	290	22.03	24.1201	25.42	Not successful	Successful	Successful
3	1	291	21.49	33.5466	18.22	Successful	Not successful	Successful
3	1	292	26.5302	33.9554	29.28	Successful	Not successful	Successful
3	1	293	negative	36.33	30.11	Not tested	Not successful	Not successful
3	1	294	21.35	30.7494	23.76	Successful	Successful	Successful
3	1	295	15.7435	23.5002	17.71	Successful	Successful	Successful
3	1	296	negative	negative	31.52	Not tested	Not tested	Successful
3	1	297	17.0432	29.1359	23.04	Successful	Successful	Successful
3	1	298	20.3838	25.7969	22.37	Successful	Successful	Successful
3	1	299	22.4444	22.2299	25.12	Successful	Successful	Successful
3	1	300	24.3345	25.1629	26.17	Successful	Successful	Successful
3	1	301	19.71	26.4016	21.76	Successful	Successful	Successful
3	2	362	negative	33.45	26.65	Not tested	Not tested	Not tested
3	2	363	negative	35.3	30.66	Not tested	Not tested	Not tested
3	2	364	negative	34.25	27.72	Not tested	Not tested	Not tested
3	2	365	negative	29.12	27.56	Not tested	Not tested	Not tested
3	2	366	negative	negative	23.44	Not tested	Not tested	Not tested
3	2	367	negative	35.74	33.1	Not tested	Not tested	Not tested
3	2	370	negative	negative	33.73	Not tested	Not tested	Not tested
3	2	372	negative	negative	35.31	Not tested	Not tested	Not tested
3	2	373	negative	negative	35.2	Not tested	Not tested	Not tested
3	2	382	negative	30.48	29.83	Not tested	Not tested	Not tested
3	2	383	negative	34.62	30.85	Not tested	Not tested	Not tested
3	2	384	negative	34.82	30.39	Not tested	Not tested	Not tested
3	2	386	negative	33.67	26.21	Not tested	Not tested	Not tested
3	2	388	negative	negative	35.34	Not tested	Not tested	Not tested
3	2	395	negative	negative	35.34	Not tested	Not tested	Not tested
3	2	397	negative	negative	35.33	Not tested	Not tested	Not tested
3	2	398	negative	negative	30.86	Not tested	Not tested	Not tested
3	2	400	negative	negative	34.76	Not tested	Not tested	Not tested
3	2	402	negative	negative	33.05	Not tested	Not tested	Not tested
3	2	405	negative	negative	27.11	Not tested	Not tested	Not tested
3	2	409	negative	negative	35.14	Not tested	Not tested	Not tested
3	2	411	negative	negative	33.31	Not tested	Not tested	Not tested
3	2	413	negative	negative	33.03	Not tested	Not tested	Not tested
3	2	415	negative	negative	32.23	Not tested	Not tested	Not tested
