# Supplementary material for: Postmortem Sampling in Piglet Populations: Unveiling Specimens Accuracy for Porcine Reproductive and Respiratory Syndrome Detection
Source: Pathogens. 2024 Aug 2;13(8):649. doi: 10.3390/pathogens13080649 (PMC11356954; doi:10.3390/pathogens13080649)
Supplement: Supplementary file 1 [file pathogens-13-00649-s001.zip › 2024 07 27 Table S1.pdf]

Table S1. Agreement between specimens by sampling point.

Specimens	Overall	F1V1	F1V2	F2V1	F2V2	F3V1	F3V2
Serum x OS	87.10%	96.67%	87.88%	75.00%	94.12%	83.87%	84.75%
Serum x NS	85.48%	93.33%	87.88%	81.25%	94.12%	77.42%	83.05%
Serum x RS	84.95%	90.00%	81.82%	93.75%	94.12%	83.87%	79.66%
Serum x TTF	67.39%	63.33%	62.50%	75.00%	93.75%	74.19%	59.32%
Serum x SIL	88.89%	NA	NA	NA	NA	90.32%	88.14%
OS x NS	92.35%	96.67%	84.21%	82.35%	100%	93.55%	95.00%
OS x RS	88.78%	93.33%	84.21%	70.59%	100%	93.55%	88.33%
OS x TTF	78.24%	66.67%	66.67%	100%	89.47%	90.32%	75.00%
OS x SIL	93.41%	NA	NA	NA	NA	93.55%	93.33%
NS x RS	88.27%	90.00%	84.21%	88.24%	100%	93.55%	83.33%
NS x TTF	75.65%	63.33%	61.11%	82.35%	89.47%	90.32%	76.67%
NS x SIL	90.11%	NA	NA	NA	NA	87.10%	91.67%
TTF x SIL	75.82%	NA	NA	NA	NA	83.87%	71.67%

F1V1: Farm 1 Visit 1, F1V2: Farm 1 Visit 2, F2V1: Farm 2 Visit 1, F2V2: Farm 2 Visit 2, F3V1: Farm 3 Visit 1, F3V2: Farm 3 Visit 2, OS: Oral swab, NS: Nasal swab, RS: Rectal swab, TTF: Tongue tip fluid, SIL: Superficial inguinal lymph node, NA: not available.
